# Male Infertility: The Effect of Natural Antioxidants and Phytocompounds on Seminal Oxidative Stress

**DOI:** 10.3390/diseases5010009

**Published:** 2017-03-01

**Authors:** Malik Adewoyin, Muhammad Ibrahim, Ramli Roszaman, Muhammad Lokman Md Isa, Nur Aizura Mat Alewi, Ainin Azwani Abdul Rafa, Mohd Nur Nasyriq Anuar

**Affiliations:** 1 Department of Nutrition Sciences, Kulliyyah of Allied Health Sciences, International Islamic University Malaysia (IIUM), Bandar Indera Mahkota, 25200 Kuantan, Pahang, Malaysia; malik.adewoyin@yahoo.com (M.A.); aizura.alewi@gmail.com (N.A.M.A.); ainaz0308@gmail.com (A.A.A.R.); nasyriq_anuar@live.com (M.N.N.A.); 2 Department of Obstetrics and Gynaecology, Kulliyyah of Medicine, International Islamic University Malaysia, Bandar Indera Mahkota, Jalan, 25200 Kuantan, Pahang, Malaysia; roszaman@iium.edu.my; 3 Department of Basic Medical Sciences, Kulliyyah of Nursing, International Islamic University Malaysia, Jalan Hospital Campus, 25100 Kuantan, Pahang, Malaysia; lokman@iium.edu.my

**Keywords:** spermatozoa, reactive oxygen species, oxidative stress, antioxidants

## Abstract

Defective sperm function has been identified as the most common cause of infertility. The objective of this study was to review recent findings on the effects of various antioxidants on male fertility. High amounts of poly unsaturated fatty acid are found in the mammalian spermatozoa membranes, thereby making them susceptible to lipid peroxidation. Although, free radicals and reactive oxygen species (ROS) play major roles in reproduction, they are strongly associated with oxidative stress. Furthermore, factors such as obesity, inflammation, pollutants and cigarette smoking are negatively correlated with spermatogenesis. Endogenous antioxidants system exists to mediate these damages. In a normal physiological state, the seminal plasma contains antioxidant enzyme mechanism that is capable of quenching these ROS as well as protecting the spermatozoa against any likely damage. However, high level of ROS triggered by inflammatory cells and oxidation of fatty acid in obese subjects may down play antioxidant mechanism resulting in oxidative stress. Evaluation of such oxidative stress is the first step in the treatment of male infertility through administration of suitable antioxidant. Notably, antioxidant such as vitamin E and C, carotenoids and carnitine have been found beneficial in restoring a balance between ROS generation and scavenging activities. There are emerging evidences that herbal products can also boost male reproductive functions. Nonetheless, a good lifestyle, regular exercise, avoidance of stress and observing safety rules at work are habits that can reverse male infertility.

## 1. Introduction

Couples begin to show concern if conception cannot be achieved after 12 months of regular unprotected intercourse. About 15% of couples desiring pregnancy are in this state globally and male factor infertility is responsible in not less than 50% of the cases [1,2]. Female partners have been deemed responsible for infertility despite the progress made by Scientist in Human reproduction in the last century. Not until the last four decades when research findings associated roughly 50% of childlessness to male factor infertility, women have been wrongly stigmatized for inability to conceive [3,4]. Essentially, factors identified in sperm dysfunction and male factor infertility are mainly environmental, physiological, and genetic [5]. No doubt, semen analysis remains the first step in the diagnosis of male infertility. However, it is quite frightening to realize that failure is being recorded in one out of five cases. ‘Male factor’ infertility is generally seen as an alteration in sperm concentration and/or motility and/or morphology in at least one sample of two sperm analyses, collected between 1 and 4 weeks apart [6]. Oligozoospermia, a medical condition characterized by low sperm count and quality is responsible for 90% of male infertility [7,8]. Notwithstanding, research findings have shown that not all men that demonstrate normal parameters in routine semen analysis are fertile. The hidden factor has been oxidative stress (OS) which is now recognized as an important and probable cause of idiopathic male infertility [9]. The fact that sperm contains a large amount of unsaturated fatty acid makes it prone to oxidation. Spermatogenic cells with oxidatively damaged DNA undergo apoptotic elimination through p53-dependent and independent mechanism which can lead to infertility. Likewise, recent data has shown that disorders such as poor fertilization, pregnancy loss, birth defects, poor embryonic development and even childhood cancer are correlated with high susceptibility of spermatozoa to oxidative insult [10,11].

This review focuses on oxidative stress as a consequence of inflammation, obesity and other sources of reactive oxygen species (ROS) as well as its impact on physiology and integrity of a sperm cell. Furthermore, the article examines the role of antioxidants and phytocompounds as mediators of oxidative stress and sperm dysfunction.

## 2. Oxidative Stress

Oxidative stress is a manifestation of excess oxidants or reactive oxygen species (ROS) against a depleted antioxidant defense mechanism in cells. The excess oxidants may be due to specific or non-specific reaction with neighboring cellular components like unsaturated lipids, DNA and proteins [12]. Sperm cells perform optimally requiring a controlled level of free radicals which is generated in the respiratory chain. In fact, the spermatozoa cannot acquire its fertilizing capacities without a small amount of ROS. For instance, in the process of capacitation, there are marked increases in the levels ROS, intracellular calcium and tyrosine kinase resulting in increased cyclic adenosine monophosphate (cAMP). Upregulated cAMP enhances the motility of spermatozoa, a condition commonly referred to as hyperactivation. However, only capacitated spermatozoa display hyperactivated motility and go through a physiological acrosome reaction, thereby acquiring the ability to fertilize. Additionally, it has been widely reported that incubation of low concentration of hydrogen peroxide with spermatozoa stimulated sperm capacitation, hyperactivation, acrosome reaction and oocyte fusion. Apart from hydrogen peroxide, nitric oxide and superoxide have also demonstrated capacity to energize capacitation and acrosome reaction [13]. Nonetheless, excessive production of ROS will not only impair its functions enumerated above such as capacitation, hyperactivation, oocyte fusion and fertilization but will result in cell damage, lipid peroxidation and DNA fragmentation [14,15].

There is a need to review WHO recommendation which limits sperm parameters to concentration and motility only. Obviously, molecular structure and sperm content is equally significant in determining the ability of a sperm to produce a healthy pregnancy. According to several studies, sperm DNA integrity is crucial for successful fertilization and normal embryonic development. Moreover, sperm oxidative stress has been associated with reduced sperm motility, excessive DNA damage, reduced acrosome reaction and decreased implantation rates in in vitro fertilization [16,17,18].

## 3. Origin of ROS

It is nearly impossible to have a ROS-free human ejaculate. Human semen contains various types of cells including mature and immature spermatozoa, round-shaped cells reminiscent of different stages of spermatogenesis, epithelial cells and leucocytes [19]. However, evidences have shown that leucocytes and spermatocytes are the major sources of ROS. Excess residual cytoplasm or cytoplasmic droplets explain the relative connectivity between increased ROS production and poor sperm quality. In fact, study has shown that cytoplasmic droplets due to defects in spermiogenesis are main sources of ROS. During spermatogenesis, a defective cytoplasmic extrusion process results in the release of spermatozoa from germinal epithelium accompanied by surplus residual cytoplasm. The released spermatozoa are mostly immature and functionally defective [20].

**Figure 1:**
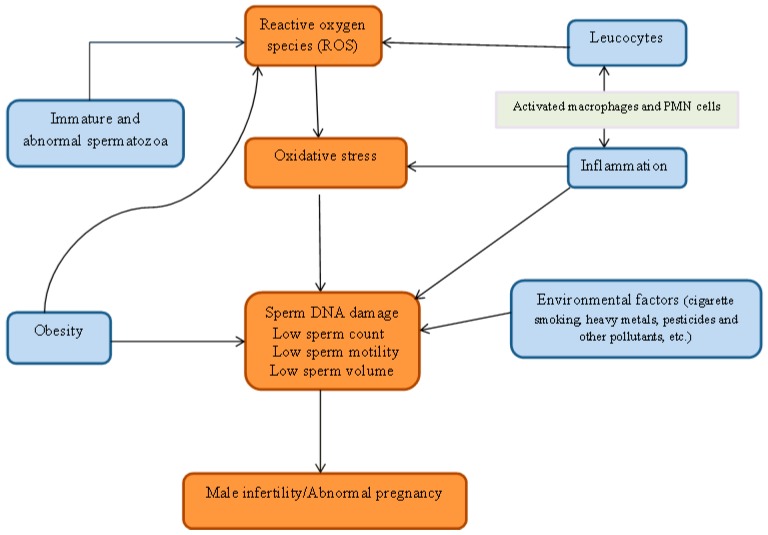
Relationship between reactive oxygen specie, oxidative stress and male infertility.

Aitken (1997) suggested a significant positive correlation between retention of cytoplasmic droplets by spermatozoa and ROS generation through a mechanism that may be assisted by the cytosolic enzyme, glucose-6-phosphate dehydrogenase [21]. Two enzyme systems have been proposed to be responsible for ROS production. They are mitochondrial oxidoreductase and the oxidase in sperm plasma membrane. Both enzymes are dependent on sperm-specific NADPH [22,23]. Essentially, ROS in human ejaculates emanate from either immature, morphologically abnormal spermatozoa or seminal leucocytes. A regular supply of energy is needed by the spermatozoa for motility hence their richness in mitochondria. Consequently, dysfunctional mitochondria stimulate increased ROS production with a resultant negative effect on its metabolic functions. This may be caused by ROS damage to the membrane while the weak mitochondrial membrane stimulates increased ROS production [24]. Similarly, studies have shown that ROS levels in fertile men are lower than in semi-fertile men. The correlation between oxidative stress and rising leucocyte count had been observed in earlier studies. It can therefore be concluded that the presence of leucocyte is connected with oxidative stress and may impair fertility as shown in Figure 1 [13,25].

Apart from macrophages that constitute about 30%, seminal leucocyte is largely polymorphonuclear (PMN) cells. Leucocyte or white blood cells are activated in response to stimuli during infection and inflammation, and these activated cells can generate up to 100-fold higher amount of ROS in comparison with non-activated cells. The activation of myeloperoxidase system of PMN cells and the macrophage leads to a respiratory burst which elevates ROS production. Sperm damage by ROS generated through leucocyte occurs if seminal leucocyte concentrations are exceptionally high such as the case in leucocytospermia [13,26].

## 4. Environmental Pollutants

The World has witnessed a decline in sperm counts over the last five decades. Studies conducted between 1930 and 1991 were based on parameters such as mean sperm density and mean seminal volume. An objective analysis of the results showed 20% drop in seminal volume and a significant 58% decline in mean sperm density from 1940 to 1990. Similarly, there are additional evidences that semen quality has also dropped considerably in the last 20 years which suggest a positive correlation between semen production and quality. It has been proposed that the sudden decline might not be unconnected with exposure to chemicals, radiation, heat and heavy metals. Additionally, DNA fragmentation during spermatogenesis can be influenced by environmental estrogen and pesticides. The toxic effect of heavy metals can also be a factor in male infertility. Constant exposure to lead for instance, without safety measures, predisposes such individuals to low fertility. Studies have confirmed that high amount of lead can hamper the sperm in performing its foremost functions, i.e., binding and fertilization of the egg. Another prominent environmental pollutant is bisphenol—A (BPA) which is used in food packaging and the production of industrial materials. BPA is a known endocrine disruptor possessing weak estrogenic and anti-androgenic activities. Findings have shown that it can impair male reproductive functions. According to a recent in vitro study on mouse spermatozoa, BPA concentration of 100 µM had a negative effect on motility, acrosome reaction, fertilization and embryonic development [27,28,29].

Furthermore, cigarette smoke is a common somatic cell carcinogen and mutagen, and may adversely affect male reproduction factors. Quite number of studies have confirmed the debilitating effect of smoking on sperm quality most especially sperm concentration, morphology and motility. Close et al. (1990) in a study on sperm penetration concluded that poor sperm function can be associated with cigarette smoking. Likewise, paternal smoking has a positive correlation with a major proportionate increase in spermatozoa with DNA damage [30]. Decline in sperm profile over the decades is strongly correlated with industrial development. It is an undisputable fact that the more we adopt a lifestyle that impairs normal physiological processes, the more the alterations in the synthesis of important hormones and proteins including the spermatozoa. Several analyses of industrial wastes have confirmed the presence of heavy metals in high concentrations. However, few industrialists adhere to prescriptions of the regulators thereby exposing workers to poisonous chemicals, radiation and other risks.

## 5. Inflammation, Oxidative Stress and Male Fertility

Inflammation is a natural host response to microbial attack or tissue injury which ultimately results in restoration of tissue vasculature and functions [31,32]. Neutrophils are the first immune cells to reach the site of infection but macrophages play a major role in inflammatory response. High amount of prostaglandins PGE2, cytokines and nitric oxide (NO) are secreted by the macrophages and other stimulated inflammatory cells [32]. However, inflammation has been suspected to affect steroidogenesis and spermatogenesis. Sharp decreases in blood levels of testosterone and luteinizing hormone has been associated with inflammation [33,34]. In a study in which inflammation was stimulated by lipopolysaccharide (LPS), a significant decrease in testosterone levels was observed. This was attributed to low activity of a major regulator of steroidogenesis commonly referred to as Steroid Acute Regulatory (StAR) protein [35].

Likewise, there are evidences that inflammation enhanced spermatogenic arrest and inhibit processes of sperm maturation. Meanwhile, cell specificity effect of inflammation is difficult to explain. While spermatocyte and spermatids are the main targets of inflammation, spermatogonia seem to be spared [36]. Epididymis is another tissue that is affected by inflammation but it is not certain whether the epididymis is targeted or it is as a result of transferred effect of testicular attack. Essentially, inflammatory response is mediated by leucocytes which infiltrate the semen and secrete anti-sperm antibodies. Inflammatory reactions enhance rigidity of sperm flagella membrane by reducing the lipid component of the membrane. Sperm motility is thereby decreased causing sperm agglutination and astherozoospermia. Resultant defects in acrosome reaction incapacitate the sperm in penetrating the oolema. Also, sperm DNA integrity is compromised resulting in increased number of apoptotic sperm [37].

Nevertheless, evidences that suggest a link between inflammation and oxidative stress in the semen are many. Infertile men exhibiting high levels of ROS on semen are mostly diagnosed with a high level of pro-inflammatory cytokines and leucocyte invasion in their semen [38]. Although, some foreign pathogens such as bacteria may generate ROS themselves, leucocytes are perceived as the most important source of seminal ROS [25]. There are two ways in which leucocytes raise the levels of ROS, i.e., direct and indirect. They do so indirectly by producing inflammatory cytokines that enhance the levels of ROS. On the other hand, significant amount of ROS are produced directly when phagocytes are stimulated and phagocytosis begins. These ROS react with spermatozoa membrane and the attack leads to oxidative burst where the ratio of oxidants to antioxidants is very high. This condition of oxidative stress persists even after the pathogen has been removed [34].

## 6. Obesity and Infertility

Obesity is caused primarily by an imbalance between energy consumed and energy utilized. In other words, overweight and obesity are defined as accumulation of abnormal or excess fat that may impair health. The commonest and simplest way to measure obesity is through the determination of BMI (Body Mass Index). Other methods are weight hip ratio (WHR), skinfold measurement, waist circumference and bioelectric impedance analysis. BMI is the ratio of weight to height that is commonly used in classifying overweight and obesity in individuals and adult population. A BMI above to 25 Kg/m^2^ is considered overweight while a figure greater than 30 Kg/m^2^ is categorized obese. Obesity is associated with several chronic disorders such as non-insulin dependent diabetes mellitus, cancer, high cholesterol, heart disease, hypertension, Sleep apnea and renal failure [39].

However, obesity is being studied as a further comorbidity factor. An appreciable quantity of data has confirmed a relationship between obesity and subfecundity. Fundamentally, obesity influences fertility and male reproductive system through its negative impact on erectile dysfunction and semen parameters [40]. Altered sperm parameters have been correlated to BMI in several studies. In a recently conducted survey, infertility in obese men was reported to be three times higher than in males from families with idiopathic cases or female – factor infertility. In addition, total sperm count and sperm density have been statistically correlated with increasing BMI. However, when WHR was used to measure obesity instead of BMI, the similar tendency of negative correlation between obesity and sperm parameters was not found. So, the inconsistency may be due to measurement techniques [41,42]. Nevertheless, evidences abound that impaired spermatogenesis and altered sperm parameters such as decreased total sperm count and concentration are significantly connected to obese men. Undoubtedly, it can be a factor in subfertility or infertility of couples. It may be suggested that obesity stimulates semen abnormalities through the production of ROS, dysregulation of hypothalamic-pituitary gonad (HPG) axis and physical manifestations [43].

## 7. Natural Antioxidants and Spermatogenesis

Generally, antioxidants are compounds that characteristically dispose, scavenge and halt the production of ROS or neutralize their actions. The main antioxidants are vitamin A, tocopherol or tocotrienols (Vitamin E), Vitamin C, beta-carotene and trace minerals. Some food supplements like selenium, Zinc, carthinine, arginine and vitamin B-12 have been shown to increase sperm count and motility. However, antioxidants such as vitamin C, Coenzyme Q, vitamin E and glutathione have been reported to be beneficial in the management of male infertility [44]. Humans have developed a highly organized and complex antioxidant defense system to shield the body’s cells and organ system from ROS. The system involves a synergy between various endogenous and exogenous components to douse the effect of free radicals [30,45].

**Figure 2:**
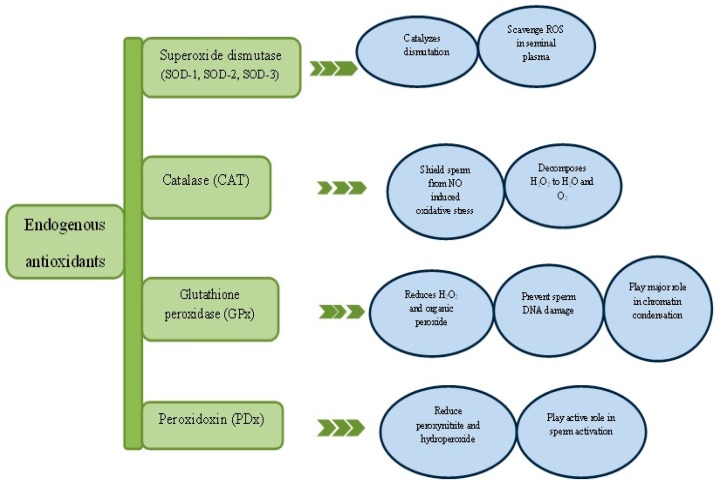
Critical role of antioxidant enzymes in spermatogenesis. ROS—Reactive oxygen species, NO—Nitric oxide.

The endogenous antioxidants (Figure 2) are basically enzymatic and non-enzymatic antioxidants like superoxide dismutase (SOD), catalase (CAT), glutathione peroxidase (GPx) and glutathione (GSH) etc. Nonetheless, exogenous antioxidants are vitamin C and E, polyphenols and carotenoids derived mainly from diets. An interaction between endogenous and exogenous antioxidants preserves and restores redox homeostasis [30,46]. A good example is the regeneration of tocopherol (vitamin E) and glutathione (GSH) or vitamin C to thwart lipid peroxidation reaction [47]. The major antioxidant in the semen is referred to as enzyme triad consisting SOD, CAT and GPx as mentioned earlier. Additionally, a relatively new family of antioxidant enzyme called peroxidoxin will also be discussed.

Superoxide dismutase (SOD) or superoxide oxidoreductase catalyzes dismutation reaction of the superoxide anions. Otherwise known as metaloenzymes, they exist in both extra- and intracellular forms. The two intracellular forms are distinguished by the metal(s) in their active centers and the organelle where they are localized. The first intracellular form contains copper and zinc (SOD-1) in the active center and is localized mostly in the cytoplasm while the second form which is found in the mitochondria with manganese in the active center is known as SOD-2. The extracellular form of SOD functions in the extracellular space (SOD-3). It is associated with the surface polysaccharide although it can be found in a free form. There are similarities between SOD-3 and SOD-2 in construction, but SOD-3 has an active center consisting of zinc and copper instead of manganese. High activity of SOD has been reported in seminal plasma with 75% of its action being associated with SOD-1 while 25% of activity has been related to SOD-3. Studies have shown that these two isoenzymes are likely derived from the prostate [48,49].

Glutathione peroxidase (GPx) plays its catalytic role by reducing hydrogen peroxide and organic peroxides which include peroxides of phospholipids. Selenium is found in the active site of GPx in the form of selenocysteine [50]. It is localized in the mitochondria matrix of the sperm but recently a nuclear form of GPx has been associated with sperm DNA protection from oxidative damage. The nuclear form has also been reported to play some role in chromatin condensation. The presence of GPx in the seminal plasma is suggestive of the fact that its source might be the prostate [51].

Another enzyme of antioxidant system is catalase which decomposes hydrogen peroxide to oxygen and water. Characteristically, it has a structure of heme system with iron atom in the center. Its activity has been reported in different organelles such as peroxisomes, endoplasmic reticulum, mitochondria and the cytosol in various types of cells. Catalase protects the cell from nitric oxide induced oxidative stress by stimulating sperm cell capacitation through a complex mechanism using hydrogen peroxide [52].

## 8. Peroxidoxins

Peroxidoxins (PRDXs) are highly expressed in most living species. They are SH dependent acidic protein having a molecular weight of 20–31 kDa. PRDXs contain one or two cysteine (cys) residues in their active site and are free of heme or selenium [53]. They form a complex with thioredoxins (TRX) reductase system to enhance their capacity to reduce both organic and inorganic peroxynitrite and hydroperoxides [53,54,55]. Because of their SH group, PRDXs target H_2_O_2_ directly and are easily oxidized in cells having low levels of H_2_O_2_. The main role of PRDx as scavengers of H_2_O_2_ is basically due to their distribution in many cellular compartments (cytosol, nucleus, endoplasmic reticulum, mitochondria and plasma membrane) [56,57]. Interestingly, studies have shown that a similar distribution pattern has been observed in human spermatozoa. At least two family members of PRDXs family members are expressed in each subcellular compartment. This differential distribution across all compartments highlights their significance in sperm as key protectors against oxidative damage. It is noteworthy that PRDX6 is highly ubiquitous and it is the only family member that is present in all organelles of human spermatozoa reacting with H_2_O_2_ at levels that trigger sperm capacitation. On account of this, it has been suggested that PRDX6 may play a critical role in the mediation of sperm activation [58].

## 9. Exogenous Antioxidants

### 9.1. Carnitines

Carnitines are polar compounds that are highly ubiquitous in nature. Dietary and endogenous biosynthesis are the two main sources of fulfilling its human requirement and the highest concentrations of carnitine in the male genital tract are in the epididymis and spermatozoa [59,60,61]. Admission and utilization of fatty acid is facilitated within the mitochondria by the carnitine which boosts cellular energetics. Besides, it restores phospholipid profile of mitochondria membrane by inhibiting fatty acid oxidation. For instance, the spermatozoa make use of the energy provided by carnitine and acetylcarnitine in sperm metabolism which impacts positively on the overall spermatogenic process. Carnitines act to safeguard the sperm and cell membrane against ROS-induced DNA fragmentation and apoptosis. Studies have shown that sperm quality and function is enhanced with a regular intake of carnitine and acetylcarnitine. In fact, low levels of carnitine have been suggested as one of the contributing factors for sperm disorders such as azoospermia and asthenospermia [61].

### 9.2. Vitamin E

Vitamin E is a very vital antioxidant molecule localized in the cell membrane. It is suggested that it inhibit lipid peroxidation and scavenge free radicals produced in the course of univalent reduction of molecular oxygen and during normal activities of oxidative enzymes. Production of these radicals results in peroxidation of phospholipid in the sperm mitochondria which culminates in low motility [62]. There are possibilities that vitamin E improves the synthesis of scavenging antioxidant enzymes. Suleiman et al. (1996) discovered that vitamin E supplementation can significantly reduce lipid peroxidation in seminal plasma, improve sperm motility and higher pregnancy occurrence [63,64]. Likewise, in a study in which a combined therapy of vitamin E and selenium were administered for six months, there was a significant increase in sperm motility and a reduced percentage of defective spermatozoa compared to pre-supplementation period [65,66].

### 9.3. Vitamin C

Vitamin C is a six-carbon keto-lactone which is biosynthesized in the liver. However, inability of humans to synthesize this essential vitamin makes it necessary to be included in the diet or as a supplement [67]. Vitamin C functions as a cofactor for various key enzymes. It helps in the metabolic processes of folic acid, tyrosine and tryptophan [68]. Vitamin C is popularly known for its role in tissue growth and wound healing [69,70]. Moreover, it has a high potency for scavenging ROS [71,72]. In a study involving 30 infertile but healthy men, daily supplementation of 200 mg and 100 mg vitamin C increased sperm count by 112 and 140 percent respectively. It is interesting to note that its concentration in the seminal plasma is 10-fold higher than the serum [73,74]. Obviously, it is a protector of human spermatozoa against oxidative damage by nullifying the effect of hydroxyl, superoxide and H_2_O_2_ radicals [75]. Semen samples with excess ROS are correlated with very low concentration of vitamin C [76]. Additionally, a combined action of vitamin C and E has been found to shield the spermatozoa against peroxidative attack and DNA fragmentation [77].

### 9.4. Carotenoids

Carotenoids are naturally occurring in fruits and vegetable. They are responsible for the yellow, red and orange pigment in plants [78]. They are important for photosynthesis and they regulate the amount of light plant is exposed. Nonetheless, humans cannot synthesize carotenoid and they rely on fruits and plants to satisfy their needs [78,79]. The most important compound in the carotenoid family is lycopene. Among the carotenoids, lycopene ranks as one of the higher quencher of singlet oxygen but a combination of carotenoids is more potent than individual compounds [80]. Outcome of research investigation has shown that lycopene is concentrated in the testes than any part of the body. This might be connected with its antioxidative role in spermatogenesis [81]. Similarly, lycopene has been found in low amount in human seminal plasma of infertile men [82]. Moreover, lycopene supplementation was also found to improve sperm motility in broilers. In the study that lasted 17 weeks, 6% increase in viability was recorded. This indicates that lycopene plays a role in maintaining sperm integrity [83,84].

Without any doubt, good diet is a key element to improving fertility. While endogenous enzymes are synthesized in the cells and tissues to counter ROS production, exogenous enzymes are derivable from plants and are capable of stimulating the production of endogenous enzymes. They can be taken as supplements to treat oxidative stress and reverse infertility. These enzymes play significant roles in maintaining the physiology of the sperm. For instance, vitamin C and E protect the sperm from DNA damage while Carnitines energizes the sperm.

## 10. Herbal Remedy for Male Infertility

### 10.1. Eurycoma longifolia Jack

**Figure 3:**
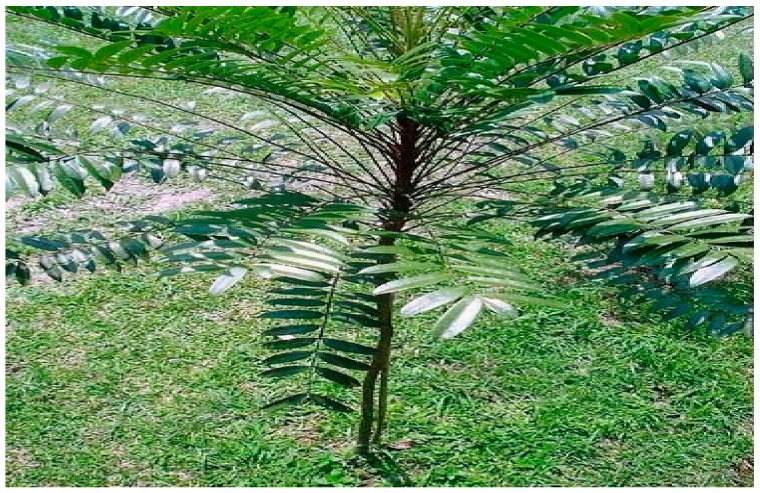
Image of *Eurycoma longifolia* jack plant. Source: [93].

Locally referred to as Tongkat Ali(picture shown in Figure 3) *Eurycoma longifolia* (EL) is native to South East Asia and belongs to the family Simaroubaceae. Malaysian EL has been found to be richer in phytochemical compounds than other South East Asia countries such as Indonesia, Thailand and Vietnam. The variations may be connected with the characteristics of the land [85]. The roots of EL contain various phytochemical compounds including quassinoids, quassinoid diterpenoids, alkaloids, eurycomaoside, eurycolactone and eurycomalactona [86,87]. Traditionally, the plant is indicated for a wide range of activity such as antimalarial, anticancer, antibacterial and male infertility [88]. Many authors have reported the capacity of the plant to boost serum concentration of testosterone [89,90]. Recently, it was demonstrated in an in vivo study that EL extract has both androgenic and pro-fertility effect. The study further dispelled the rumor that the plant might not be safe [91]. In a similar report, water soluble extract of EL was found to overcome symptoms of late-onset hypogonadism and related disorders [90]. Earlier studies have indicated that eurypeptide which is a compound found in EL is capable of stimulating the biosynthesis of various androgens [90,92].

### 10.2. Cardiospermum halicacabum

**Figure 4:**
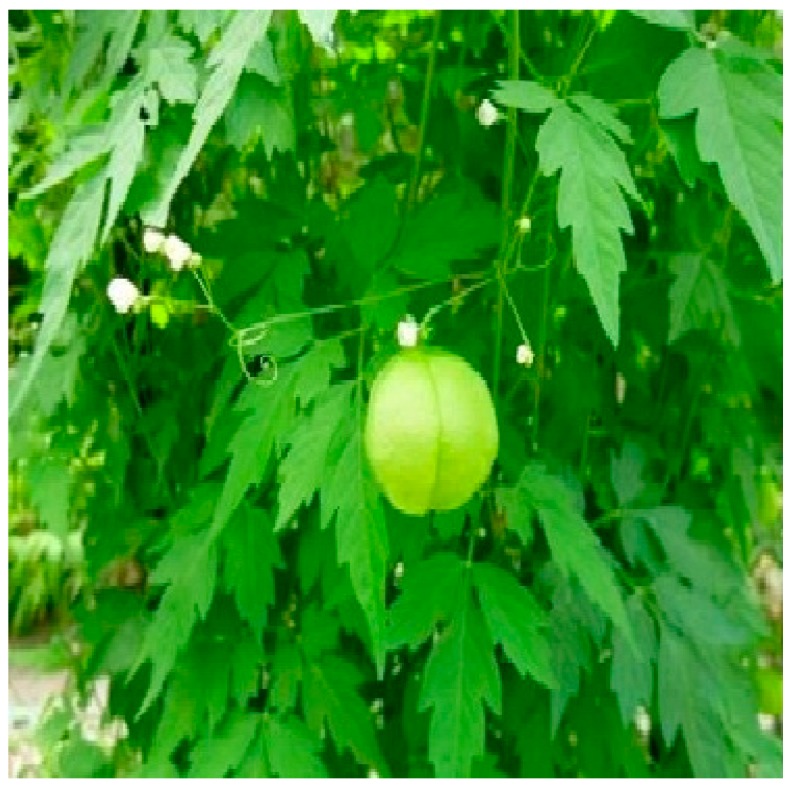
Image of *Cardiospermum halicacabum* plant. Source: [96].

*Cardiospermum halicacabum* (CH) is popular among tradomedical practitioners in Sri Lanka. It is used to treat rheumatism, snake bite and bleeding piles [94]. However, the plant which is commonly referred to as balloon vine has been found to increase fertility in male Wister rats. After 30 days administration of CH, a significant increase in caput and epididymal sperm count as well as sperm motility was observed. The plant also boosted serum testosterone level which is associated to Saponin in CH. The effect of CH ( picture shown in Figure 4) on sperm parameters may be as a result of its broad spectrum of phytocompounds most especially flavonoids that are known for their antioxidative properties [95].

### 10.3. Grape Seed Extract

**Figure 5:**
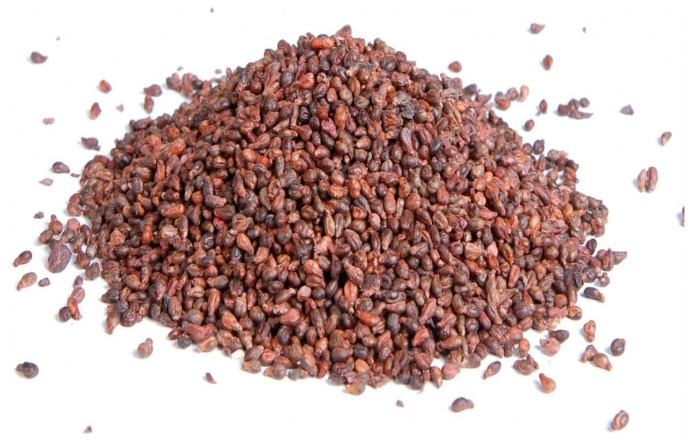
Image of grape seed. Source: [102].

Grapevine (*Vitis vinifera*, shown in Figure 5) grows in all temperature regions of the world. Grape seeds extract have been reported for antiinflammatory, antioxidant and antimicrobial activity. It has cardioprotective, hepatoprotective, and neuroprotective effects as well [97]. Studies have shown that grape seed contain a particular flavonoid called anthocyanin oligomers in considerable amounts. This compound increases intracellular vitamin C levels and scavenge ROS and free radicals. In fact, it has a greater antioxidative activity than vitamin C and vitamin E [98,99]. Grape seed extract increased sperm count, viability and sperm motility in a study in which testicular dysfunction was induced by aluminium chloride. Similarly, it protected the sperm cell against DNA damage. The extract prevented nitric oxide (NO) invasion of the testis by reducing the activities of nitric oxide synthase [100]. Grape seed has also been reported to attenuate apoptosis of germ cells induced by torsion/detortion of the testicles [101].

### 10.4. Marjoram Essential Oil

**Figure 6:**
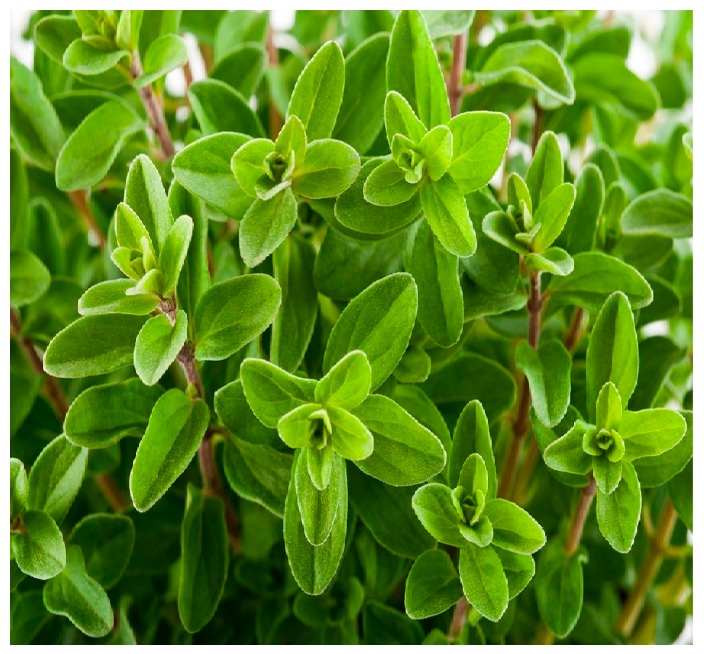
Image of Marjoram plant. Source: [106].

Generally, dried leaves of Marjoram (*Origanum majorana*) and its flower tops are natural sources of Marjoram. *O. majorana* (OM) contain many bioactive compounds including flavonoids, terpenoids, sisterol and phenolic glycosides [103]. In folk medicine, marjoram extracts are used for cramps, coughs, dizziness, depression, gastrointestinal disorders and migraine [99]. Marjoram (picture shown in Figure 6) has displayed capacity to increase both spermatogenic and sperm cells in an experiment in which degenerative changes in seminiferous tubules were induced by high fat diet. There were improvements in lipid profile in serum and testis as well as increase in androgens. In contrast, a decline in weight, adiposity index, leptin and prolactin levels were observed. Likewise, sperm count and the testicular structure were comparable with that of the normal group [104]. In a related study, a synergistic action of OM and grape seed extract on reproductive function was evaluated by using ethanol to induce oxidative stress and reproductive disturbances. Ethanol increases lipid solubility of cell membranes thereby changing the permeability of blood-tissue barriers and ultimately allowing more xenobiotics to access different organs. Administration of ethanol reduced weights of testis, epididymis and sex organs which were restored by both OM and grape seed extract. There was also a significant increase in the levels of serum testosterone in animals treated with the combined formulation [105].

### 10.5. Syzygium aromaticum

**Figure 7:**
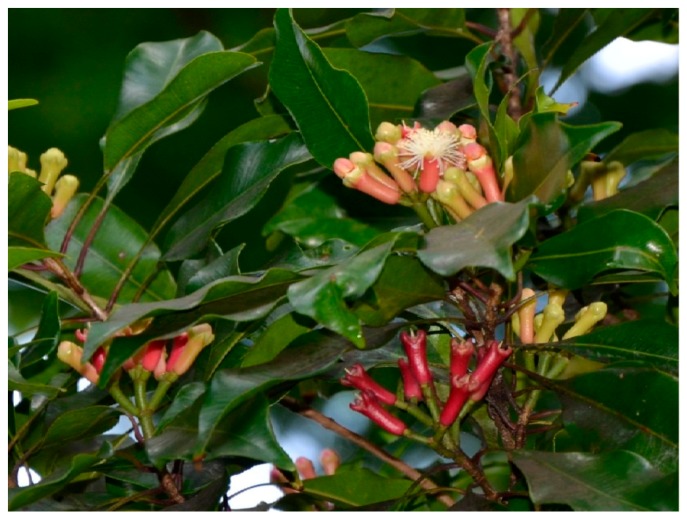
Image of *Syzygium aromaticum* plant. Source: [109].

*Syzygium aromaticum* (SA) of Myrtaceae family is native to Indonesia and it is commonly referred to as clove. It is a tree of small or average size having a height of 8–12 m. Although, SA (shown in Figure 7) is native to Indonesia, the plant is well recognized in Australia and South East Asia as food flavor and remedy for ailments such as dental disorder, headache and respiratory diseases. Traditionally, SA has been an age long cure for sexual dysfunction and low libido [107]. Boudou et al. (2013) used an overdose of manganese chloride to stimulate reversible infertility in Wistar rats. There was a significant reduction in body weight and testis in negative groups that are exclusively given manganese chloride. The manganese group was also characterized with a degeneration of seminiferous tubules, absence of sperm or low sperm count, large interstitial space and deficient Leydig and basement membranes. In contrast, the histological sections of the seminiferous tubules of the group treated with SA are richly populated, appear healthy and signs of regeneration are manifest [108].

### 10.6. Nigella sativa

**Figure 8:**
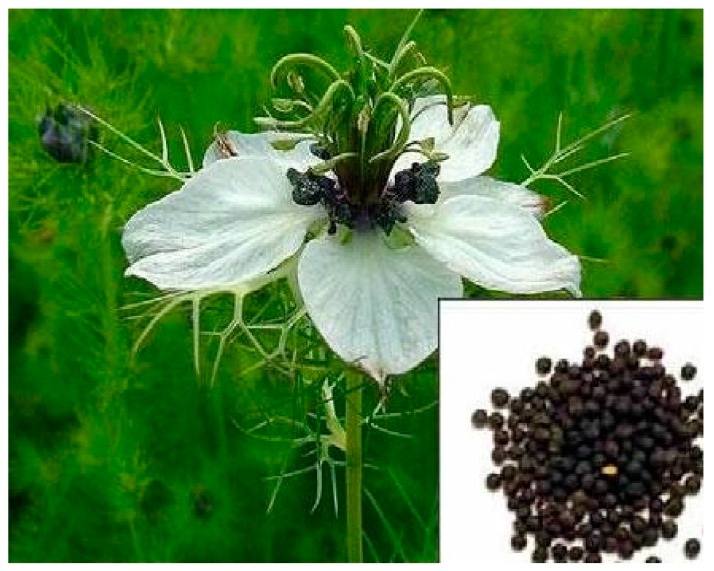
Image of *Nigela sativa* plant. Source: Pure Life 2017 [111].

*Nigella sativa* (NS, shown in Figure 8) is widely grown in the South of Mediterranean and the Middle East. Analyses of the seeds show that it contains more than 100 compounds. The spicy seeds have been used in cooking pastries and curries over the ages and the oil is exclusively reserved for medicinal purpose. Nonetheless, studies have shown that the seeds have antiviral, antiinflammatory and immunomodulatory activities. Haseenah et al. (2015) demonstrated the effect of NS on spermatogenesis using streptozotocin induced diabetic rats. At the end of the study, testosterone and luteinizing hormone were expectedly low in diabetic rats while the groups treated with NS had a significant increase in the level of testosterone. Diabetic men have been diagnosed with sub fertility characterized with reduced sperm motility and concentration as well as increased abnormal morphology [110].

### 10.7. Lycium barbarum

**Figure 9:**
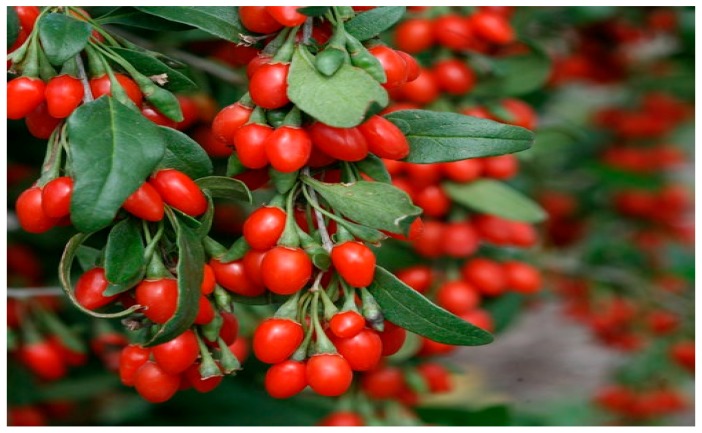
Image of *Lycium barbarum* plant. Source: [113].

*Lycium barbarum L*. (picture shown in Figure 9) is a member of plant family called Solanaceae. Traditionally, Red-colored fruits of *Lycium barbarum* have been used for curative purposes by Chinese herbalist for thousands of years [112]. However, Luo et al. (2006) decided to verify a 16th century claim that the fruit has pro-fertility effect. For more than four centuries, Li Shizhen claim has given the *L. barbarum* a fair share of aphrodisiac market in Chinese societies. Luo et al. (2006) chose to use *L. barbarum* polysaccharide by removing lipids and oligosaccharides through refluxing and filtering the powdered fruit in appropriate mixed solvent system. In the study, 36 rats were used to assess the protective effect of LBP on organs of reproductive system after 24 h exposure to heat (43 °C). Apart from this, six male Kong Ming mice were sacrificed and their testicular cells isolated for in vitro studies. Testicular cells pretreated with different concentrations of LBP were induced with hydrogen peroxide to stimulate DNA damage. The group further tested the effect of LBP on sexual behavior of rats in a separate study involving 46 males and 46 females.

At the end of study, following observations were made: degenerative signs in the heat exposed testis, irregular seminal tubules, disappearance of spermatids and sperms as well as so many abnormalities in the spermatocytes. However, biochemical and histological data show partly restoration of morphological integrity of seminiferous tubule in the testis of rats treated with LBP. Furthermore, DNA damage induced in testicular cells was clearly attenuated by different doses of LBP. The DNA chains were broken in the untreated group. Similarly, the sexual behavior of the treated animals improved upon the administration of LBP. There was also an increase in the level of testosterone [114].

### 10.8. Tribulus terrestris

**Figure 10:**
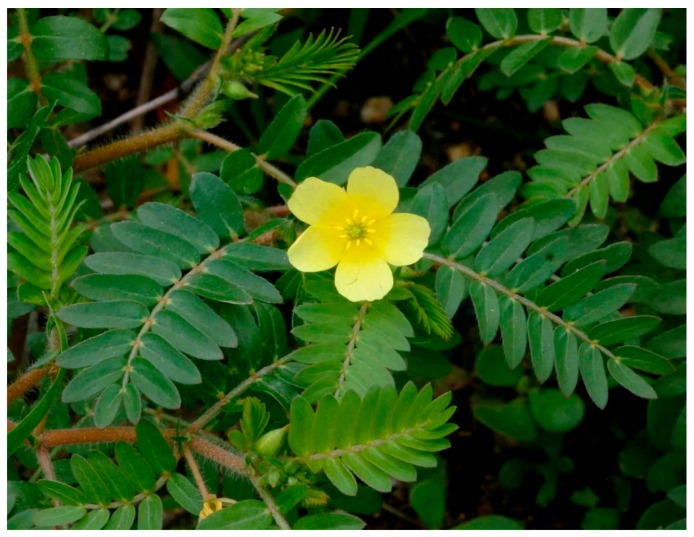
Image of *Tribulus terrestris*plant. Source: [119].

*Tribulus terrestris* (shown in Figure 10) plant, commonly referred to as puncture vine is a widely distributed perennial creeping herb [115]. *T. terrestris* extracts have been used in tradomedical practices to treat common ailments such as inflammations, edema and ascites [116]. The plant has long been identified as a cure for treating male infertility in Europe and Asia [117]. Recently, protective and antioxidant effect of methanolic extract of *T. terrestris* fruits (METT) was evaluated using rats stimulated with sodium valproate (SVP). The chemical is capable of inducing testicular toxicity and oxidative stress. The rats in the negative control group that received only sodium valproate had decreased weight in testes and seminal vesicles. Biochemical test showed low levels of serum testosterone, FSH and LH. Low semen quality and quantity were also observed. The action of the SVP also affected levels of antioxidant enzymes such as SOD, GPx and CAT. Histopathological sections of testes show edema, necrosis and marked atrophic seminiferous tubules. Nevertheless, the administration of METT increased the weight of the testes and seminal vesicles. It also improved semen quality and quantity in a dose dependent manner. Likewise, it increased the levels of testosterone, FSH and LH. Treatment with METT effected a partial amelioration of Histopathological lesions [118].

### 10.9. Asteracantha longifolia

**Figure 11:**
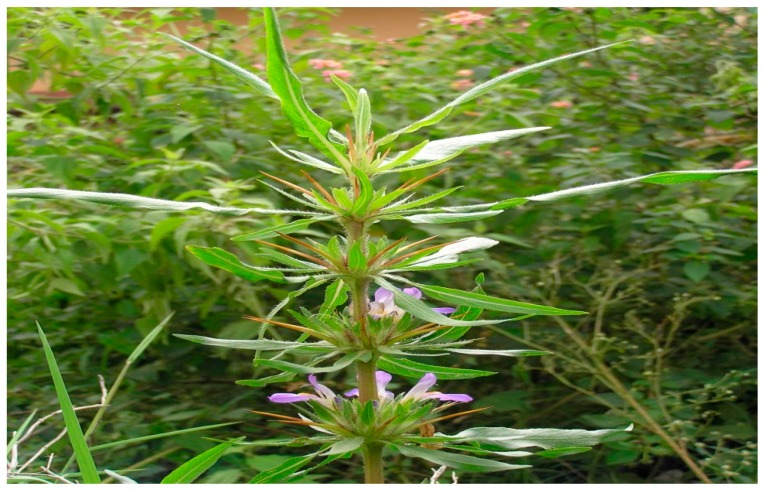
Image of *Asteracantha longifolia* plant. Source: [123].

*A. longifolia* (shown in Figure 11) belongs to the family Acanthaceae and is known since the ancient times in India for its medicinal values. The roots of *A. longifolia* have served as cure for diarrhea, dysentery, poor libido and anaemia [120]. Similarly, Chauhan et al. and Sahu et al. (2010) reported the potency of *A. longifolia* (AL) seeds as androgenic and aphrodisiac agents. However, a team of researchers in India (2015) underwent a study on the ability of AL to protect the testis of rats induced with toxic dose of cadmium chloride [121,122].

Administration of CdCl2 increased the thickness of the interstitial space in the negative control group as compared to the normal control. There was a significant improvement in the diameter of seminiferous tubules in rats that were administered CdCl2 and AL seed powder concurrently. Additionally, AL seed powder increased height of sertoli cells and reduced the increased thickness of interstitial space due to CdCl2 toxicity. All Stages of germ cell suffered a significant decrease in diameter when treated exclusively with CdCl2. In contrast, *A. longifolia* seed powder ameliorated the effect of Cdcl2 toxicity by increasing the micrometric measurements of spermatogonia, primary and secondary spermatocytes as well as spermatids [124]. Studies have shown that cadmium decreases testosterone production and distorts regulatory mechanism of hypothalamic pituitory-gonadal axis [125,126].

### 10.10. Polycarpea corybosa

**Figure 12:**
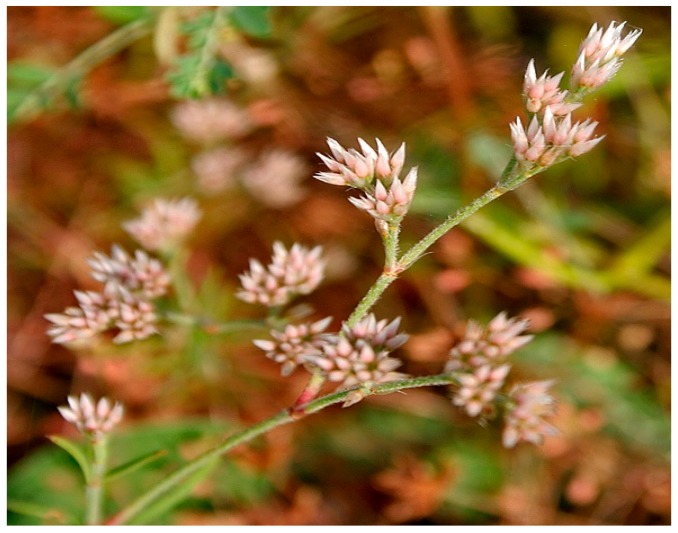
Image of *Polycarpea corybosa* plant. Source: [129].

*Polycarpea corymbosa* (picture shown in Figure 12) is popular among the natives of Sirumalai hills, Western Ghat Tamil Nadu. Locally referred to as Pallipoondu, *P. carymbosa* is well known for its antiinflammatory and hepatoprotective activities [127]. Recently, ethanol extract of *Polycarpea corymbosa* was reported to have boosted sperm motility and density while it reduced sperm abnormality. Furthermore, the whole plant extract of *P. carymbosa* increased serum levels of testosterone and LH compared to normal group. There was also a significant increase in females impregnated by male rats administered *P. carymbosa* in contrast with untreated rats. In addition, the effect of the plant extract stimulated increases in the weight of testes, epididymis, vas deferens, ventral prostate and seminal vesicle [128].

## 11. Discussion

Essentially, the effect of medicinal plants (Table 1) on male reproductive functions is associated with antioxidant activity [108,130]. In other words, the potential of phytomedicines to improve male fertility is a function of amount of antioxidants found in the plant. Furthermore, studies have shown that antioxidants improve various processes of male reproductive function such as spermatogenesis and steroidogenesis [131,132]. Characteristically, all the plants discussed in the previous section are all antioxidants. For instance, LBP protected the mouse testicular cells against H_2_O_2_-stimulated DNA damage in a dose dependent manner. The possible mechanism of scavenging the hydroxyl group is either through a suppression of lipid peroxidation of testicular cells thereby reducing DNA damage of the cells; or through activation of antioxidant enzyme system to attenuate DNA damage. Antioxidant enzyme system is a naturally designed structure found in most cells to avert ROS-induced injury [133,134].

Reduction of oxidative stress levels may boost natural conception and the outcome of assisted reproductive technologies. Antioxidants provide the most critical defense against free radical induced male infertility. Nevertheless, infertile male subjects with high levels of ROS are identified through standard semen analysis and by determining sperm deformity index [135]. Additionally, direct methods such as chemiluminescence assays, flow cytometry, cytochrome-c and nitroblue tetrazolium reduction, xylenol orange-based assay and electron spin resonance spectroscopy can also be used to quantify seminal fluid oxidative stress. Biomarkers of oxidative stress such as DNA damage, thiobarbirutic acid reactive substances, total antioxidant capacity and isoprene can be measured as an indirect method of determining the level of ROS in the subjects under examination.

However, the future of semen analysis may lie in the field of proteomics. This field is fast expanding because of advances in two-dimensional electrophoresis (2-DE) for protein separation and mass spectrometry (MS) for peptide sequencing. Enzyme-linked immunosorbent assay and Western blotting are some of the proteomics technique that are used to confirm the proteins spotted in 2-DE while signaling pathways associated with sperm fertility are determined through Pathway Studio software. In a recent study, 20 differentially expressed proteins were identified in boar spermatozoa. Some of the proteins included mitochondria trifunctional protein, GPX4, glutathione-S-transferase (GSTs), pyruvate dehydrogenase, Ras related proteins (Rab-2A), arginine vasopressin receptor 2, etc. The study discovered new signaling pathways to understand interactions between identified proteins and others at the cellular level. GPX4 and Rab-2 were found to be associated with capacitation whereas GST and some other proteins were connected with structure of spermatozoa and stress response. The authors believe that the proteins identified in the study may be useful as negative biomarkers for detection of inferior male fertility]. Nonetheless, understanding the mechanism and significance of tyrosine phosphorylation will deepen our knowledge of sperm functions and male fertility. Several studies have suggested that sperm motility, hyperactivation, capacitation, the acrosome reaction, chemotaxis, and sperm-zona pellucida-binding are regulated by tyrosine phosphorylation. Since some of these processes can be directly or indirectly affected by ROS species, tyrosine phosphorylation may be an important factor in future research on the effect of oxidative stress on male fertility [136,137,138,139,140,141].

Furthermore, measuring the level of oxidative stress should be a prerequisite in antioxidant supplementation so as to avoid wrong medication. Both natural and synthetic antioxidants have gained attention of industries such as nutritional, pharmaceutical and cosmetic. However, their relevance in reproduction and fertility management is still in infancy stage. The non- enzymatic antioxidants highlighted in a section above like vitamin E, C, carotenoids and carnitine may shield the spermatozoa from oxidative DNA and membrane damage by reducing singlet oxygen and lessening the detrimental effect of lipid peroxidation on sperm [4,76]. For instance, Pentoxifline is an artificial sperm motility stimulator as well as a scavenger of ROS. Undoubtedly, the therapeutic use of these antioxidants appears promising, further controlled clinical studies are needed to determine if majority of these putative antioxidants can actually help patients overcome infertility or at least improve sperm viability of the patients [4].

**Figure 13:**
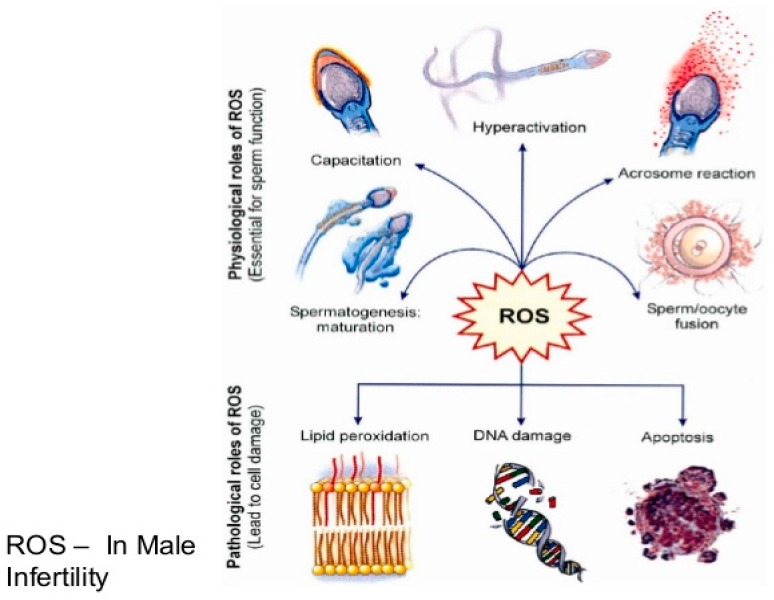
Contradictory role of ROS in spermatogenesis. Source: [142].

After establishing the need to administer antioxidants, dose of antioxidant preparations should be looked into critically. In case of oxidative stress, higher than normal daily doses should be taken for a minimum of three months because, it takes spermatogonia 72 ± 4 days to transform to a mature sperm [47]. A six-month treatment with Vitamin E alone has been reported to improve semen concentration and motility. Likewise, in a similar study, a significant increase in sperm motility was observed after a three-month administration of vitamins A, E, C and selenium. It can therefore be suggested that the antioxidant could be administered for three to six months [139]. In as much as lower doses may not give a desirable effect, uncontrolled doses may induce infertility by significant reduction of ROS production (as depicted in Figure 13) as observed by Kwon and his team recently. The team established a correlation between excessive generation of glutathione peroxidase (GPx4) and infertility due to inhibition of ROS production. GPx4 is not only an important antioxidant enzyme for sperm motility; it plays a critical role prior to capacitation. However, while GPx4 prevents sperm apoptosis, it suppresses ROS which is an essential factor for capacitation [137].

**Figure 14:**
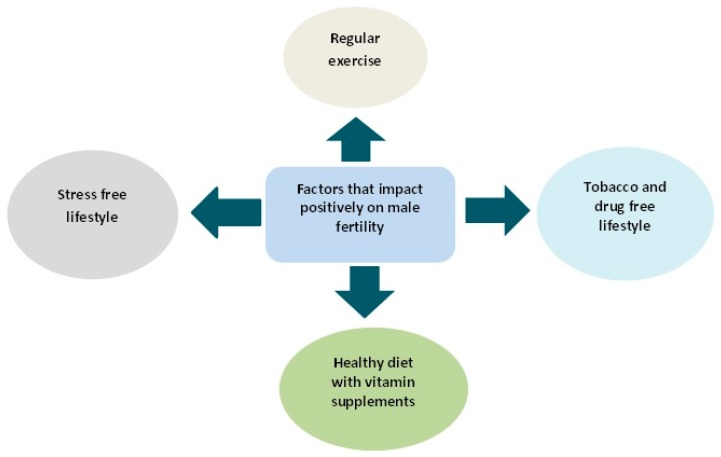
Fertility boosting lifestyle.

Emerging scientific evidences are in support of negative impacts of obesity on fertility, sperm function and even the health of offspring. However, life style changes in terms of diet and regular exercise can reverse not only the disease state but the offspring outcome (Some of the factors that boost fertility are shown in Figure 14). In a recent study, intake of selenium fortified probotics by obese rodents boosted their metabolic health and sperm profile (sperm count and motility) [143]. Similarly, diet and exercise intervention in obese mouse confirmed a positive correlation between sperm function and metabolic health of male subjects. Successful reversal of plasma concentration of insulin, glucose and cholesterol to normal levels will result in overall improvement in metabolic health and consequently improve sperm motility and morphology as well as reduction of oxidative stress and DNA damage [17].

Lastly, exposure to heat, toxins, pollutions and heavy metals contribute to development of oxidative stress. Any activity that may trigger an increase in scrotum’s temperature such as extended driving, hot bath, saunas, and long office hours should be avoided. Workplaces should be adequately aerated and safety rules adhered to strictly [37].

## 12. Conclusions

Oxidative stress is a common phenomenon in the aetiology of many diseases and male infertility is not an exception. Reactive oxygen species play a significant role in spermatogenesis and reproduction. However, certain physiological conditions which may be induced by inflammation, obesity or toxins exacerbate the production of these species culminating in sperm DNA damage. A number of defense mechanism including antioxidant enzymes, vitamins (E, C and carotenoids) and biomolecules are critical in the living system. Essentially, normal functioning of the spermatozoa may require a balance between ROS and antioxidant. Although, natural antioxidants and phytocompounds have demonstrated beneficial role in spermatogenesis, a thorough diagnosis is required to determine patients that need to be supplemented. Nevertheless, healthy living, regular exercise and stress free jobs may help reverse sperm dysfunction.

## Figures and Tables

**Table 1:** Selected plants and their effect on male fertility.

Common Name	Botanical Name	Effect on Fertility	Other Medicinal Applications	References
Tongkat Ali	*E. longifolia*	Prevents hypogonadism, stimulates biosynthesis of androgens	antimalarial, anticancer, antibacterial	[90,91,144,145,146,147]
Baloon vine	*C. halicacabum*	Enhances caput and epididymal sperm count, sperm motility and serum testosterone	Rheumatism, bleeding piles and snake bite	[94,95,148]
Grapevine	*V. vinifera*	Improves sperm profile; Protect sperm from DNA damage; alleviate apoptosis of germ cell	Antiinflammatory, antioxidant, antimicrobial, hepatoprotective	[100,101,149]
Marjoram	*O. marojana*	Increases sperm cells and androgens; protect sex organs; increases serum level of T	For treating cramps, coughs, dizziness, depression etc.	[104,105]
Clove	*S. aromaticum*	Cure for sexual dysfunction and low libido	Treatment of dental disorder, headache and respiratory diseases,	[107,108,150,151]
Black seed	*N. sativa*	Enhances levels of T and L hormone	For cooking, antiviral, antiinflammatory, immunomodulatory	[110,152,153,154,155]
Wolfberry	*L. barbarum*	Protect sperm cells from DNA damage; increases serum T	General medicine	[156,157,158,159,160,161]
Puncture vine	*T. terrestris*	Increases weight of testis and seminal vesicle and serum level of T	Cure for inflammation, edema, ascite	[117,118,147,162,163,164,165]
Hygrophila	*A. longifolia*	Increases weight of sertoli cells; enhances micrometric measurement of spermatogonia, spermatocytes and spermatids	Cure for diarrhea, dysentery and anemia	[121,122,124,166,167]
Pallipoondu	*P. carymbosa*	Increases levels of T and L hormone; enhances weights of testis, epididymis and vas deferens	Antiinflammatory, hepatoprotective	[128,168,169]
